# Emerging roles of TRIO and F-actin-binding protein in human diseases

**DOI:** 10.1186/s12964-018-0237-y

**Published:** 2018-06-11

**Authors:** Sungjin Park, Hyunji Lee, Minhee Kim, Jisoo Park, Seon-Hwan Kim, Jongsun Park

**Affiliations:** 1 Department of Pharmacology, Metabolic Syndrome and Cell Signaling Laboratory, Institute for Cancer Research, College of Medicine, Chungnam National University, Daejeon, 35015 Republic of Korea; 2 Department of Neurosurgery, Institute for Cancer Research, College of Medicine, Chungnam National University, Daejeon, 35015 South Korea; 3 Department of Medical Science, College of Medicine, Chungnam National University, Daejeon, 35015 Republic of Korea

**Keywords:** TRIOBP, Hearing loss, Cancer, Actin-binding protein, Actin cytoskeletal organization, Tara

## Abstract

TRIO and F-actin-binding protein (TRIOBP) also referred to as Tara, was originally isolated as a cytoskeleton remodeling protein. TRIOBP-1 is important for regulating F-actin filament reorganization. TRIOBP variants are broadly classified as variant-1 or − 4 and do not share exons. TRIOBP variant-5 contains all exons. Earlier studies indicated that TRIOBP-4/5 mutation is a pivotal element of autosomal recessive nonsyndromic hearing loss. However, recent studies provide clues that TRIOBP variants are associated with other human diseases including cancer and brain diseases. In this review, recent functional studies focusing on TRIOBP variants and its possible disease models are described.

## Background

TRIO and F-actin-binding protein (TRIOBP) was originally identified as a cytoskeleton-associated protein that prompts actin cytoskeletal reorganization and cell proliferation and migration [1]. To date, TRIOBPs have been studied as two distinct variants, TRIOBP-1 or TRIOBP-4/5. In this review, the categories of three major TRIOBP variants for human diseases and subcellular functions are discussed as well as recent associations between TRIOBP and cancer.

### TRIOBP variants

**Fig. 1:**
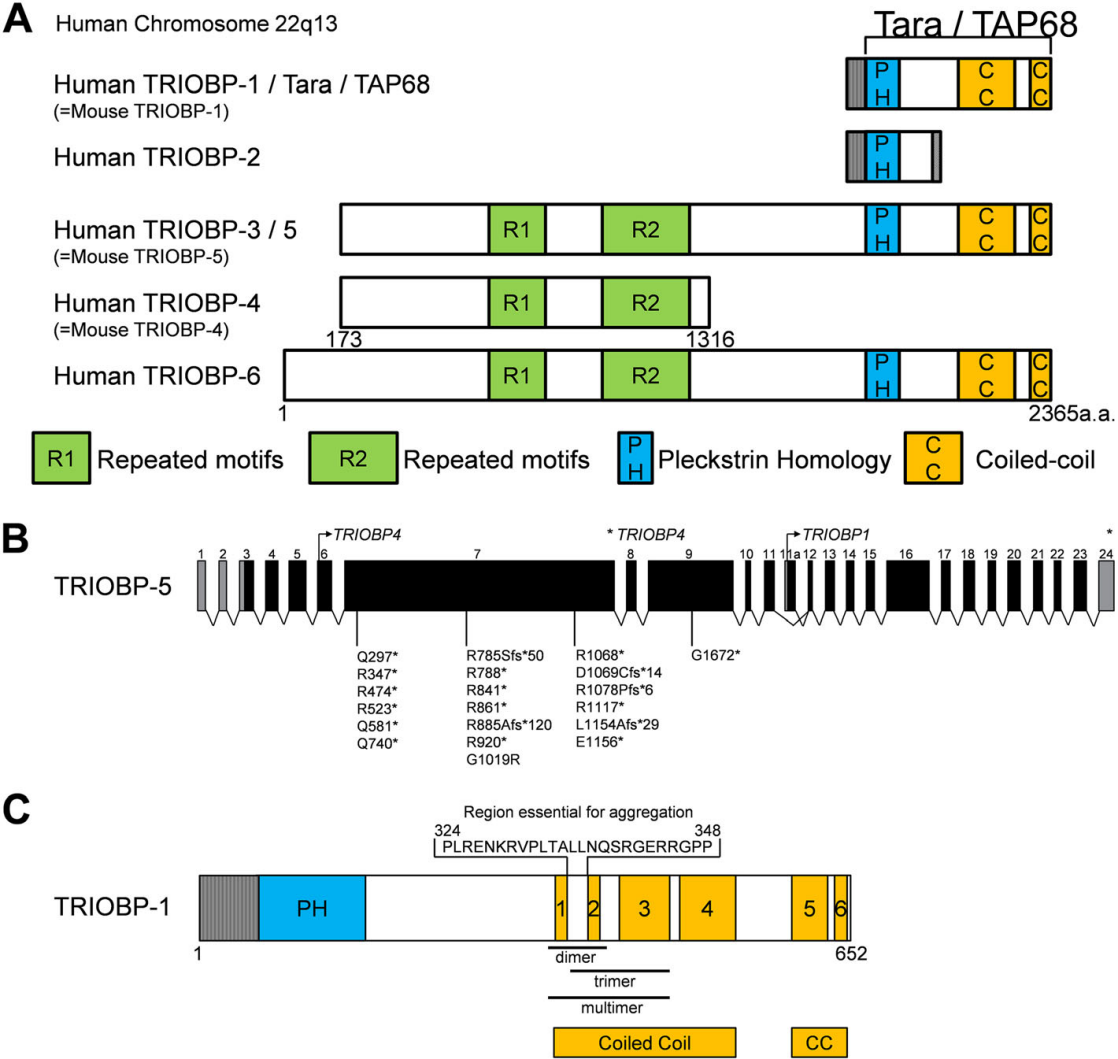
Schematic Representation of TRIOBP Variants. **a** Structure of TRIO and F-actin-binding protein (TRIOBP) variants via alternative splicing. *TRIOBP-1* and *TRIOBP-4* do not share any genetic sequences. **b** Genetically deafness frequently was mutated in exons 6–9 of TRIOBP-4. **c** The first and second coiled-coil regions contain multimerization sites in TRIOBP-1

TRIOBP is expressed through two promoters and can be classified into three variants (Fig. 1a). The first variant is TRIOBP-5 (218 kDa), the longest transcript in humans, the first promoter that encodes from exon 1 to exon 24. The second variant is TRIOBP-4 (107 kDa), a shorter protein product, that contains the repeat motifs of exon 6 but none of the carboxyl-terminal domains of TRIOBP-5. The third variant, represented by Tara/TRIOBP-1 (72 kDa) [1], is initiated from the second promoter downstream of exon 6. TRIOBP-1 encodes a protein that does not contain the N-terminal repeat motifs but does include the C-terminal domains of TRIOBP-5 encoded by exons 11–24. Therefore, TRIOBP-1 and TRIOBP-4 do not share any exons or amino acid sequences. Overexpression of TRIOBP-1 (also known as TAP68 or Tara) interacts with and stabilizes the actin cytoskeleton in human HeLa cells [1]. TRIOBP-1 is ubiquitously expressed in all tissues, whereas TRIOBP-4 and TRIOBP-5 are predominantly expressed in the retina and inner ear [2, 3].

### Role of TRIOBP-4/5 in hearing loss

The phenotype of Deafness, autosomal recessive 28 (DFNB28), is characterized by prelingual, severe to profound sensorineural hearing impairment. In 2006, two research groups found that DFNB28 maps to chromosome 22q13.1 and is associated with TRIOBP-4 and TRIOBP-5 (TRIOBP-4/5) mutations in hearing loss patients in 15 families [2, 3]. To date, in 22 families, all TRIOBP mutations causing human deafness are frequently located in exons 6–9 only in *TRIOBP-4/5*, but do not affect TRIOBP-1 [4–8] (Fig. 1b).

**Fig. 2:**
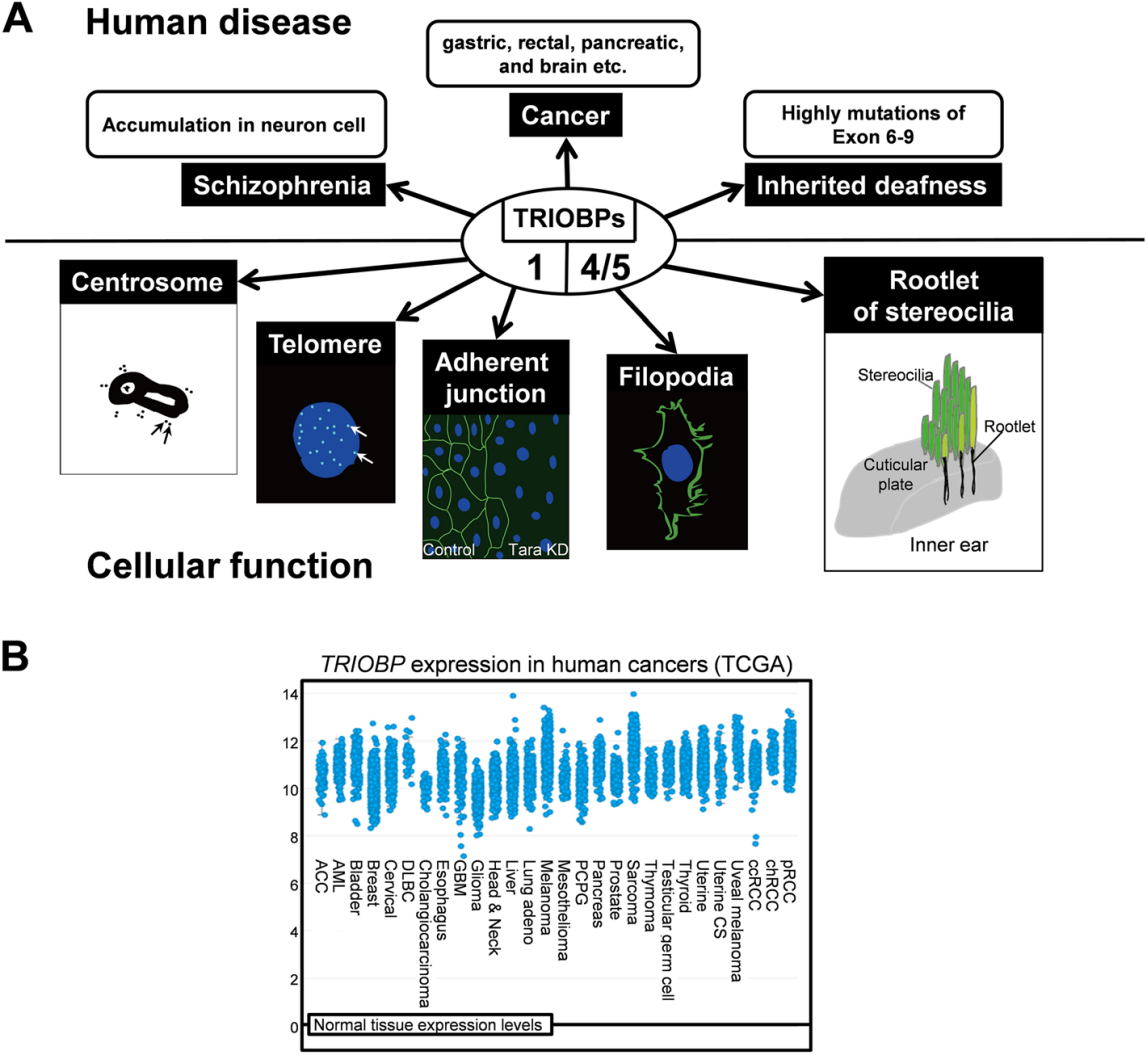
Roles of TRIOBP Variants for Human Diseases. **a** Classification of cellular functions and human diseases based on the major TRIOBP variants 1 or 4/5. **b** Human *TRIOBP* expression in different tumor types from The Cancer Genome Atlas database. Adapted from cBioPortal: http://www.cbioportal.org/index.do

Inner ear hair cells detect sound through deflection of hearing sensor stereocilia. The stereocilia are linked by rootlets in the cuticular plate of the inner ear (Fig. 2a). The C-terminus of TRIOBP-5, corresponding to coiled-coil domain of TRIOBP-1, is localized at rootlets [9]. Exon 6 of TRIOBP-4/5 encodes several copies of two repeated motifs, R1 and R2; only the R1 motif is the major actin-binding domain of TRIOBP-4/5 [2, 10]. TRIOBP-4/5 is the protein that plays a pivotal role in the formation of rootlets, because *Triobp-4* knockout mice cannot form rootlets. In addition, stereociliary fusion in both inner hair and outer hair cells was observed in *Triobp-4* knockout mice [11, 12]. The mutations of TRIOBP-4/5 in human hereditary deafness DFNB28 leads to stereociliary fusion caused by disruption of actin networks in the apical region of inner ear hair cells.

### Role of TRIOBP-1/Tara/TAP68 in disease

TRIOBP-1 (referred to as Tara or TAP68) is associated with regulating actin cytoskeletal organization. To date, the disease relevance of TRIOBP-1 is less clear than that of TRIOBP-4/5. TRIOBP-1 consists of an N-terminal pleckstrin homology (PH) domain and a C-terminal coiled-coil region which is responsible for its homo-multimerization [1, 13]. TRIOBP-1 is ubiquitously expressed in mammalians [2, 3]. To reorganize the actin cytoskeleton, TRIOBP-1 recruits Nuclear distribution element-like 1 (Ndel1) to F-actin structure [14]. Nde1 interacts with TRIOBP-1 to regulate cell migration. TRIOBP-1 is associated with telomeric repeat binding factor 1 (TRF1) and recruits TRF1 and Tankyrase to the centrosome during mitosis [15]. Thr457 of TRIOBP-1 is phosphorylated by Polo-like kinase 1 (Plk1), which plays important regulatory functions during mitosis. The centrosomal localization of TRIOBP-1 depends on the Thr457 phosphorylation. Centrosomal localization of phosphorylated TRIOBP-1 by Plk1 is important for normal chromosome segregation [16]. Moreover, TRIOBP-1 is ubiquitinated by the E3 ubiquitin ligase HECTD3. The ubiquitination of TRIOBP-1 promotes the degradation of TRIOBP-1. The degradation of TRIOBP-1 caused by HECTD3 knockdown mediates genomic instability [17]. These results indicate that regulation of TRIOBP is important for mitotic processes and the cell cycle and may be associated with cancer.

### Accumulation of TRIOBP-1 in the brain

Protein aggregations have been shown in the brain of patients with chronic mental illness caused by disruption of protein degradation, specifically, in schizophrenia studies. A monoclonal antibody was found to detect TRIOBP-1 in brain aggregomes exclusively of schizonphrenia patients, and not controls. TRIOBP-1 has a high propensity to aggregate when overexpressed in neuroblastoma cells, unlike TRIOBP-4. Endogenous TRIOBP-1 may also spontaneously form aggregomes in post-mitosis, consistently causing aggregation of TRIOBP-1 in the differentiated neurons [13, 18]. The aggregation property of TRIOBP-1 is caused by the homo-multimerization site (amino acids 324–348) between the first coiled-coil and second coiled-coil domain (Fig. 1c) [13]. These results suggest that TRIOBP-1 aggregation can lead to mental illness.

### Oncogenic TRIOBPs in Cancer

A frameshift deletion mutation of TRIOBP was found in one family with gastric and rectal cancer [19]. Although the mutation could not be determined as contributing to cancer, TRIOBP may be a hereditary factor in cancer. TRIOBP-4/5 is significantly upregulated in pancreatic cancer cells and human cancer tissues. TRIOBP-4/5 facilitates the motility of pancreatic cancer cells by regulating actin cytoskeletal reorganization in the filopodia of the cells [20]. TRIOBP-1 is also abundant in adherent junctions and regulates the organization of epithelial cell sheets and integrity by upregulating E-cadherin transcription leading to carcinogenesis [21] (Fig. 2a). Both transcripts and proteins of TRIOBP-4/5 are upregulated in glioma and glioblastoma multiform (GBM) cell lines. TRIOBP-1/5 is significantly upregulated in GBM patients. Specific siRNAs for TRIOBP-4/5 reduced cell proliferation and migration in GBM cells [22]. Interestingly, specific siRNAs for TRIOBP-1 reduced the cell migration and proliferation in human mesenchymal stem cells [23, 24]. The Cancer Genome Atlas (TCGA) analysis also confirms that TRIOBP mRNA is enhanced in most cancer patients (Fig. 2b).

Taken together, these results contribute to the understanding of TRIOBP function in broad tumors. Consequently, TRIOBPs may be a novel diagnostic marker and therapeutic target for glioma and other cancers.

### Further focus for TRIOBPs

The oncogenic function of TRIOBPs is poorly understood. Biochemical evidence is lacking to encourage studies regarding this gene. However, recent studies have shown that all TRIOBP variants may contribute to most cancers including GBM. Based on the available data regarding TRIOBPs, in this review we described and associated their characteristics with cellular biology topics, therefore providing several promising aspects for future TRIOBP studies.

Based on previous studies, TRIOBP variants 1/4/5 have different functions. Our recent study showed that all TRIOBP variants are upregulated in GBM [22]. The molecular mechanisms of TRIOBP variants that regulate tumorigenesis are unclear. Interestingly, TCGA data indicate a relationship between TRIOBPs and most cancers. Tissue-specific *TRIOBP-4/5* gene is expressed in brain cancers but not in normal brain tissues. Further studies focusing on TRIOBPs as potential cancer biomarkers or regulators of cancer metabolite are necessary [25].

The accumulation of TRIOBP-1 associated with brain diseases such as schizophrenia has been suggested. Thus, further neurobiological studies regarding the effects of TRIOBPs on brain cancer are necessary.

The cellular localization of TRIOBPs is required to determine the specific function of TRIOBPs in subcellular organelles such as centrosomes, telomeres, filopodia, and adherent junctions. In addition, understanding TRIOBP function during the cell cycle such as mitosis or interphase could provide anti-cancer strategies for tumorigenesis.

## Conclusions

The first known gene, TRIOBP, to be associated with hearing loss has recently been linked to cancer. TRIOBP variant 1 and TRIOBP variant 4 have been reported to have completely different functions, but both have been found to be associated with cancer. Finally, a new anti-cancer strategy would be identified through a study of the cancer mechanism of TRIOBP variants.
